# Perspective Commentary: The Implementation of Welfare Policies Are Not Held to the Same Ethical Standards as Research: Raising Intergenerational Health Inequality Concerns

**DOI:** 10.3389/fpubh.2021.764559

**Published:** 2021-11-23

**Authors:** Sophie Wickham, Daisy Fancourt

**Affiliations:** ^1^Department of Public Health, Policy and Systems, Institute of Population Health, University of Liverpool, Liverpool, United Kingdom; ^2^Research Department of Behavioural Science and Health, Institute of Epidemiology and Health Care, University College London, London, United Kingdom

**Keywords:** welfare policies, ethics, intergenerational, health inequalities, safety, COVID-19

## Abstract

Over the last 12 years the United Kingdom (UK) has seen the introduction of an austerity programme—a fiscal policy—with the primary goal to reduce the government's budget deficit and the role of the welfare system. Between 2010 and 2015 there was an estimated reduction of £14.5 billion in spending, attributable to decreasing the value of benefits and restricting entitlement to benefit claimants. By 2020, there had been an estimated unprecedented £27 billion less spent on welfare compared with spending in 2010. Whilst fiscally-successful at reducing spending, some implemented welfare policies have had direct consequences for people's health, increasing inequalities which have been heavily criticized. Moreover, there is growing concern that this has an intergenerational effect. In this paper, we describe the ethical principles in human research, how these have been considered in public health policy, and the existing evidence of the direct and intergenerational health and welfare consequences of some recent, nationally-implemented welfare policies. We argue that ethical principles, specifically the ethical principle of *safety* that is applied in all research, should be applied to all public welfare policies to stop the rising inequalities in health we are seeing across generations. We highlight that initial changes implemented to welfare policies as a response to COVID-19 demonstrate that there can be a political and societal perceived value in going further to support individuals and their families during times of adversity, and consider the ethical implications of this.

## Introduction

### Ethical Principles in Medical Research and Policy

Since 1964 when the World Medical Association published the Declaration of Helsinki, specific ethical principles for all medical researchers and research studies involving human participants have been in place. These have been meticulously monitored, revised and updated over the years to reflect the needs of an evolving society (1). These principles and processes are designed to safeguard individuals from mental or physical harm during and subsequent to research participation. But they arguably have a relevance to any wider activities that have the potential to cause harm to the health or well-being of individuals. For example, the Declaration of Helsinki requires physicians to make the health of the patient “their first consideration” and to act “only in the patient's interest” (quoting the Declaration of Geneva). It requires that those leading research “promote and safeguard the health, well-being and rights of patients,” ensuring their “respect.” It highlights the need to continually check and challenge treatments and tools “through research for their effectiveness, efficiency, accessibility and quality.” In particular, this involves testing the “benefits, risks, burdens and effectiveness of new interventions…against those of the best proven interventions.” Research must “never take precedence over the rights and interests of individual research subjects.” It must involve “individuals with the appropriate ethics and scientific education, training and qualifications.” Compensation “for subjects who are harmed as a result of participating in research must be ensured,” and “appropriate arrangements for post-trial provisions” must be made. These principles are checked and approved by research ethics committees before any study begins, and these committees have the right to monitor ongoing studies, receiving updates on any serious adverse events. Further, consent to participate must be voluntary, with participants receiving full details on conflicts of interest, institutional affiliations of those involved, and the risks and potential benefits. Crucially, participants can refuse to participate and withdraw from involvement later on. Further, for transparency, studies are recommended to be registered in a publicly accessible database and results to be published.

The relevance of these principles to the development and implementation of national welfare policies is clearly apparent. Welfare policies focus on addressing basic human needs such as access to food, accommodation and shelter, which are core determinants of human health and survival. Poorly-designed or implemented welfare policies have the potential to do significant harm to health, not just to individual's directly in receipt of welfare support, but their children, who are often indirectly effected through increases to household poverty. Yet despite this, in public welfare policy, there are no strict ethical principles underlying policy development and implementation.

Concerns about the *effectiveness* of implemented policy interventions have been discussed for decades. In the 1960s, a famous article entitled “Reforms as Experiments” argued that any social reform (instigation of new policies and changing or cessation of existing policies) should be routinely carried out experimentally with randomized controlled trials as a way of balancing innovation and caution (2). In the wake of this, many national policy changes, from alterations of electricity and water prices to induce consumer conservation of resources to the implementation of prison rehabilitations to prevent reoffending, have been tested experimentally (3). The development of more sophisticated experimental methodologies and bespoke guidelines for trialing complex interventions have supported such work (4). However, in the UK this has been applied inconsistently, with no penalization for policies that do not take this approach. Further, it is often the largest and most radical policy changes, where effectiveness is most essential to determine, that do not take an experimental approach.

However, arguably the main danger of this is not the challenge of assessing the *effectiveness* of policies (i.e. whether public money has been effectively spent and initial problems that led to the policy have been effectively solved); that debate has been discussed elsewhere (3–5). Instead, the main danger is whether policies are *safe*. Indeed, there have been active concerns that health and social care policies specifically, which are designed to support people in low-paid employment, unemployment, and disability, to promote inclusion, health, and well-being during times of adversity, in particular, have been ignoring ethical evidence (6).

### Examples of Ethical Issues in Recent Welfare Policies

There have been numerous ethical issues in recent welfare policies. Introduced over the last 12 years the United Kingdom (UK) has seen the introduction of an austerity programme—a fiscal policy—with the primary goal to reduce the government's budget deficit and the role of the welfare system. Between 2010 and 2015 there was an estimated reduction of £14.5 billion in spending, attributable to decreasing the value of benefits and restricting entitlement to benefit claimants. By 2020, there had been an estimated unprecedented £27 billion less spent on welfare compared with spending in 2010 (7). Whilst fiscally-successful at reducing spending, some implemented welfare policies have had direct consequences for people's health, increasing inequalities which have been heavily criticized. As one example, the Working Capabilities Assessment was introduced in 2010 as a programme designed to reassess people on out of work disability benefits with two possible outcomes: movement onto a new disability benefit (employment and support allowance) or movement off their disability benefit (and on to job seekers allowance). However, research has shown that this policy has been causally associated with suicides, self-reported mental health problems and antidepressant prescribing (8). But perhaps the most stark example in recent years has been the introduction in 2013 of universal credit (UC). Implemented by the Department for Works and Pension (DWP) as one of the largest welfare changes to the benefit system it was an entirely digital service, replacing 6 previous means tested welfare benefits for working age adults (including child tax credit, working tax credit, housing benefit, income-based job seekers allowance, income support and income-based employment and support allowance). The primary goal of introducing UC was to “encourage more people into work by introducing better financial incentives, simpler processes and increasing requirements on claimants to search for jobs” (9), thus supporting the government's fiscal policy to reduce the role of the welfare system. The government committed to assessing its implementation, but only in terms of labor market outcomes (10), despite the fact that the reform has been controversial, with doctors and third sector organizations highlighting the damaging health effects of UC (11, 12). Qualitative research has found that for claimants managing the UC process, its increased conditionality combined with the threat of sanctions exacerbated long-term health conditions and negatively affected participants' mental health such that some had considered suicide (13). A recently published study using a nationally representative longitudinal sample of over 50,000 people found a rise of 7% points in psychological distress for unemployed people affected by the UC's introduced. Though the effect size was modest, the implications are far reaching, as this is a national programme affecting many vulnerable people, with an estimated 60,000 people across the UK negatively affected between 2013 and 2018, and over 20,000 people becoming clinically depressed (14).

### The Intergenerational Health Impact of Welfare Reform

**Figure 1:**
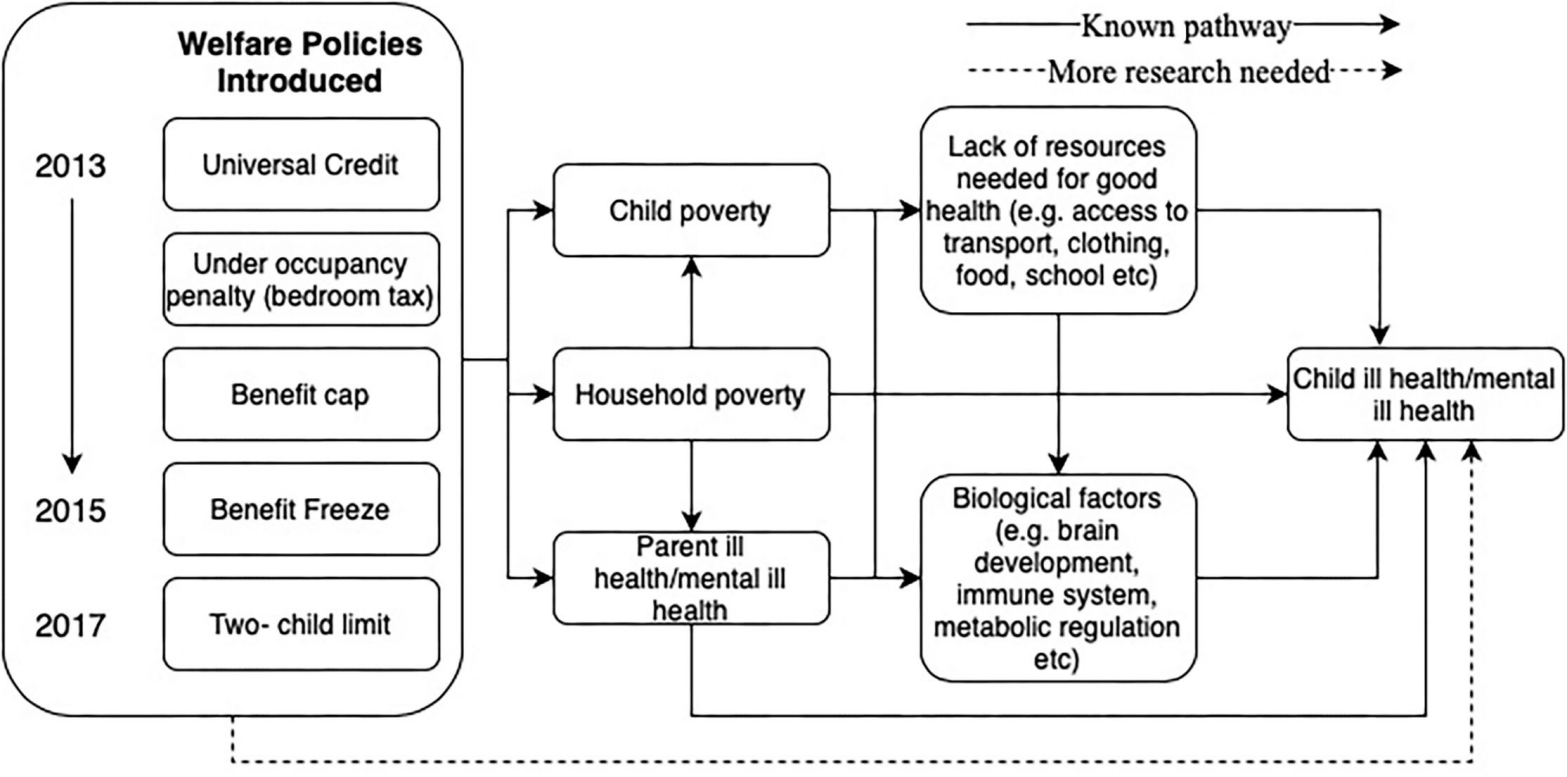
Potential pathways and mechanisms from specific welfare policy changes to poor parent and child health mental health.

Understanding the pathways from welfare reform to child health outcomes, and the possibility of widening health inequalities is of huge public health concern, as this will have direct consequences for an individual's quality of life, their ability to enter and stay in the workforce, contribute to the economy and their use of the health, social and welfare system. Unfortunately, the evidence-base for the intergenerational health impact of welfare policies is scarce. Predominately, evidence is restricted to (i) investigating the individual income (or poverty) and health impact of welfare reform (8, 14) and (ii) investigating the impact of health and income (or poverty) of adults on their children (15). Figure 1 demonstrates these potential pathways identifying known and lesser known pathways, for example, the secondary damage experienced by welfare changes that occurred prior to the pandemic that were targeted at adults have been exposed with stark consequences for child poverty rates.

Prior to 2010 child poverty rates were falling (a known risk factor for negative health and well-being outcomes for children and young people). However, after the introduction of austerity driven policies child poverty started to rise, leading many to conclude a causal relationship [see (16)] between policy changes and rising child poverty levels. The evidence base for the negative health and well-being outcomes for children and young people because of rising poverty is irrefutable (Figure 1) (15, 17–20).

There have been changes to UC and many of the existing welfare policies that have occurred as a direct response to the coronavirus disease 2019 (COVID-19) that have had adverse effects across generations. For example, following the start of the pandemic in 2020, the government increased the basic rate of UC and working tax credit by £20 a week, alongside a range of other financial support measures from relaxing the minimum income floor for self-employed (21), introducing additional council tax relief funds (22), uprating child benefit, and introducing statutory sick pay on day 1 of sickness as opposed to day 4 (23). Some of these measures have been temporary and it is arguable that some are a product of the exceptional circumstances that COVID-19 has created and might not be as applicable in ‘ordinary’ circumstances. However, the uplift to UC turned out to be vital to keep many people above the poverty line. Evidence has demonstrated that the loss of the uplift could result in 700,000 people being pushed into poverty, including 300,000 children, and 500,000 more people than were already in poverty being pushed further into deep poverty (24). Despite such evidence of this major intergenerational adversity, the uplift was removed in October 2021.This has potential to further damage the health and well-being across multiple generations if ignored.

Thus, whilst the research evidence is in its infancy, what is available is showing more and more clearly that as well as direct negative health consequences of welfare change on adults, there can also be secondary effects down the generations. Consequently, there is an urgent need to directly explore the intergeneration health effect of welfare policy changes and specifically to ask “are our welfare policies ethical?”

## Ethics in Welfare Policy

### The Importance of Considering Ethics in Welfare Policy Implementation

In exploring this question, a key consideration is how unsafe policies are implemented in the UK. Often, there is simply not enough impact evidence, or piloted information, or even that existing evidence is not followed before major national policy changes are brought in, leading to evidence-*informed* rather than evidence-*based* policy shifts (25). Consequently, the evidence gaps are often filled with good intentions but insufficient data. These may not be enough to prevent harm (5). If we look beyond welfare policies to consider interventions from other sectors, the scientific evidence is littered with examples of well-meaning interventions that caused unintentional harm. This point was highlighted in a previous discussion piece 20 years ago (5), that cited examples from bicycle safety education programmes for children that increased risk of injury (26), toughened pint glasses in bars for injury prevention that led to a rise in glass-related injuries (27), and prison visits for youth offenders intended to deter future offending that in fact increased it (28). Thus, the assumption that plausibility is a sufficient basis for decision making is acknowledged as dangerous within a broad range of sectors, but it needs to be better acknowledged within welfare policy (5). Even if there is sufficient evidence-base for a potential policy implementation, unintended negative consequences can be observed, which highlights the need for continuing monitoring impacts of policy changes. Moreover, it is possible that while an intervention has some benefits, unintended negative consequences may also be observed meaning that an intervention needs adapting. For example, if we return to UC, attempts to move people off welfare support and into work, as a primary goal of the policy and the main route out of poverty, transitioning adults into sustainable employment may have in fact resulted in harm. Early evidence suggests that moving onto UC has not resulted in people moving into employment (14). Furthermore looking at the child poverty data, the majority of children living in poverty (over 75% of children) are in households where someone is working (29). This suggests that in its current form, moving people into work does not guarantee a positive outcome. These unintended negative consequences are well-accepted as important outcomes to monitor and respond to in other sectors, especially within health research, but need to be considered more carefully within welfare policy.

There is another reason too that welfare policies should be taking extra steps to ensure that their policies are safe. Many of the studies cited above are from studies that tested the effects of interventions in research prior to large-scale implementation, or that evaluated small-scale pilots prior to roll-outs. As such, there were opportunities to identify risks and make decisions accordingly; risks were relatively contained. The roll-out of many welfare policies, such as UC, has not taken this same cautious approach. Consequently, where negative effects on health occur, they can happen fast and at scale, with little or no measures in place to mitigate their impact. It is understandable that changes in health and social outcomes relating to specific interventions are difficult, complex, and understanding the mechanisms involved are even more so, making it hard to prevent unintended consequences. However, we argue that there are currently insufficient measures in place to ensure that health impact is properly considered and monitored.

All of this is not to say that beneficial policies are scarce. Many policy developments have had wide-ranging positive effects on individuals and society. For example, the introduction of the national minimum wage, introduced to tackle poverty, has been found to positively impact individuals mental health (30). It would be advantageous to consider the intergenerational health impact of this as there are likely to have been secondary health benefits for children too. There are strong causal effects seen for both children and adults health and well-being from raising individual and household income (31, 32). But this only serves to highlight more acutely the importance of considering and monitoring health within all welfare policies: the process of changing policy does not have to have adverse effects.

Beyond the UK context, consideration of ethical standards needs to be implemented in all countries, regardless of wealth, political context or healthcare system design. Internationally, there have been shifts to consider ethical standards within policy implementation (33). However, the focus on ethical practices are largely on developing countries, and whilst welfare policy implementation may learn from methodologies used in other policy areas (e.g., health policy), it remains an absent factor.

### Potential Solutions

What this discussion highlights is the criticality of comprehensively identifying ethical issues of policies in advance, monitoring these and other unforeseen adverse effects during pilots and implementation, transparency in the development of policies and in impact assessments, as well as having the provisions to modify or halt the implementation of a policy if there is evidence of harm. Unsafe policies can emerge through lack of understanding, evidence, piloting and monitoring. These ethical considerations should apply not just to the policy itself, but also to the very process of individuals being exposed to unfamiliar regimes of processes, rules, and conditions as this exposure alone can cause distress, expense and negative effects on selfhood (34). More careful attention is needed to identify whether economic or social benefits are likely to be outweighed by negative outcomes simply from changing the status quo for individuals and their families.

We are already seeing some moves toward this approach. Research within bioethics is extending ethical debate into arenas such as public health and social care (35), whilst the development of implementation science and applied health research is providing rich insight into factors such as the acceptability, suitability and reach of interventions (36). But given the evidence coming to the fore on the adverse effects of interventions such as UC across generations, current approaches are clearly not sufficient. Of course, there is a balance to be struck between ethical policy implementation and moralistic implementation (37). Ethics for policy need to be context specific rather than being overly uniform. Further, ethical decisions on policies should not replace democratic decisions (38). But at present, we apply far stricter criteria to research studies that pilot new interventions (even low-risk interventions) usually at a small-scale than we do to the nation-wide implementation of major policy interventions, and as we are seeing, this is doing harm. Furthermore, health effects are rarely considered within welfare policy design and piloting, and there is a need for rigorous trials to tackle this. Whilst policies overall are seeing a shift toward impact assessments above and beyond what they are designed to do, welfare policy impact assessments are behind and need implementation.

In times of crisis we look to the fundamental needs of a nation and health and welfare are central to those needs. Welfare systems need to adapt to changing circumstances but they should be done in a way that does not disadvantage anyone. More than a decade of austerity programmes has contributed to the damaging health impacts we are seeing of COVID-19 across generations. Whilst we welcome the small shifts we are seeing to welfare policies in the UK, many were temporary measures to weather the storm of the current pandemic. If they are seen to be necessary to meet basic human needs now when many people are experiencing extreme hardship, why does this principle not extend to normal life when extreme hardship is still experienced, just by a smaller population? However, it is encouraging that quick implementation and changes are possible to the welfare system. Now is the time to measure and monitor the inter-generational health consequences of welfare policies, to investigate those planned, implemented and already existing, to ensure that the health and health inequalities impact is assessed and appropriate ethical considerations have been considered.

## Public Involvement

This piece describes research into the effects of welfare policies that has drawn in detail on public involvement. Whilst this analysis piece did not involve any further work on this, we will involve the public to help us develop our dissemination strategy.

## Data Availability Statement

The original contributions presented in the study are included in the article, further inquiries can be directed to the corresponding author/s.

## Author Contributions

SW and DF contributed to the drafting, interpretation, and revision of the manuscript equally. All authors approved the submitted version of the manuscript. SW's expertise is in public mental health and their work has focused on the mental health impact of poverty and welfare changes with a focus on universal credit. DF's expertise is in the physical and mental health impact of social determinants and experiences.

## Funding

SW and DF were both supported by Wellcome Trust Society and Ethics Fellowships (200335/Z/15/Z & 204507/Z/16/Z respectively). The funders had no involvement in the analysis or in the decision to submit the paper for publication. This work was also funded by the Nuffield Foundation [WEL/FR-000022583], but the views expressed are those of the authors and not necessarily the Foundation.

## Conflict of Interest

The authors declare that the research was conducted in the absence of any commercial or financial relationships that could be construed as a potential conflict of interest.

## Publisher's Note

All claims expressed in this article are solely those of the authors and do not necessarily represent those of their affiliated organizations, or those of the publisher, the editors and the reviewers. Any product that may be evaluated in this article, or claim that may be made by its manufacturer, is not guaranteed or endorsed by the publisher.
